# The Missing Axis: Cardiovascular–Kidney–Metabolic Risk After Gestational Diabetes

**DOI:** 10.34067/KID.0000001297

**Published:** 2026-07-30

**Authors:** Mengyao Tang

**Affiliations:** Division of Nephrology, Department of Medicine, Massachusetts General Hospital, and Harvard Medical School, Boston, Massachusetts

**Keywords:** CKD, diabetes mellitus

Gestational diabetes mellitus (GDM) is one of the most common metabolic complications of pregnancy. Although diagnostic criteria and screening protocols vary across jurisdictions, approximately 14% of pregnancies globally are affected, with prevalence rising in parallel with the obesity and type 2 diabetes epidemics and against a backdrop of older maternal age at first pregnancy.^1^ Most women with GDM are normoglycemic by the time of the postpartum visit, but biochemical resolution belies a durable risk trajectory. A history of GDM confers an approximately ten-fold higher risk of subsequent type 2 diabetes^2^ and a roughly two-fold higher risk of incident cardiovascular events, with the cardiovascular signal amplified further when GDM co-occurs with hypertensive disorders of pregnancy.^3^ GDM has accordingly come to be regarded as a stress test or window into future cardiometabolic disease, a framing at present embedded across diabetes, obstetric, and cardiovascular guidelines and in postpartum care frameworks. The kidney has received far less attention.

In this issue of *Kidney360*, Echouffo-Tcheugui et al. bring the kidney into focus, reporting the largest nationwide cohort study of GDM and incident kidney disease to date.^4^ Drawing on the French national hospital discharge database, the study included 1,441,317 parous women who delivered in 2012–2013, with 10 years of follow-up; 7.2% had a GDM diagnosis on the basis of International Classification of Diseases codes. A history of GDM was associated with a 46% higher adjusted risk of incident hospitalized CKD and a more modest 18% increase in AKI. When postpartum incident hypertension and type 2 diabetes were added as time-dependent covariates, the CKD signal attenuated but remained significant, while the AKI signal disappeared. The elevated CKD risk was detectable within the first year postpartum and rose monotonically over the decade. Risk was also dose dependent and scaled with the number of GDM episodes. The study’s strengths include large sample size, near-complete national capture, systematic GDM screening in a universal coverage system, and time-varying modeling of important covariates such as hypertension and diabetes. Its limitations stem largely from the claims-based design: Kidney outcomes are restricted to International Classification of Diseases-coded hospitalizations rather than laboratory-confirmed eGFR or albuminuria, so early-stage CKD is likely under-recognized and absolute incidence underestimated; body mass index is captured only at the extreme of morbid obesity; race and lifestyle factors are not available; and 10 years remains short for a disease whose natural history spans decades.

These results sit within an accumulating evidence base. A recent systematic review and meta-analysis of 11 cohort studies (21.3 million women, 7.2% with GDM) reported a pooled hazard ratio of 2.19 (95% confidence interval, 1.70 to 2.68) for incident maternal CKD after GDM.^5^ Heterogeneity across studies is substantial, reflecting differences in GDM diagnostic thresholds, follow-up duration, CKD outcome definitions, and how incident type 2 diabetes and hypertension during follow-up are handled in the analysis. A central and previously unresolved question has been whether GDM contributes to kidney injury independently or whether the kidney risk entirely stems from subsequent type 2 diabetes and hypertension, two well-established risk factors of CKD. A Swedish national cohort found that women with GDM who never progressed to overt type 2 diabetes had a risk of developing CKD indistinguishable from women with uncomplicated pregnancies, implying that downstream overt diabetes carried nearly all of the risk.^6^ By contrast, a Danish nationwide cohort used formal mediation analysis to show that diabetes accounted for 75% of the GDM–CKD association and hypertension 30% but that an excess CKD risk persisted even in women with insulin-treated GDM who never developed subsequent diabetes.^7^ The current French data add further evidence that GDM contributes to kidney injury beyond the risk it confers through later type 2 diabetes. After full adjustment for both postpartum hypertension and type 2 diabetes, a residual 10% excess CKD risk persisted.^4^

Several biologically plausible mechanisms could explain an independent effect. Pregnancy itself raises GFR by approximately 50%, and incomplete recovery of glomerular hemodynamics may follow pregnancies complicated by metabolic stress. Persistent insulin resistance, endothelial dysfunction, inflammation, podocyte injury, and metabolic memory are additional candidates. The early postpartum emergence of risk, however, is unlikely to fully reflect an acute pregnancy-induced kidney insult. It more probably reflects chronic metabolic dysfunction that predates the index pregnancy and is unmasked by the combined physiologic stress of gestation and routine antepartum screening. Put another way, pregnancy is less a cause of subsequent disease than a life event superimposed on a pre-existing cardiometabolic and kidney risk trajectory.

The study has important clinical implications. Postpartum metabolic surveillance after GDM is already well established. Both the American Diabetes Association and the American College of Obstetricians and Gynecologists recommend a 75-g oral glucose tolerance test at 4–12 weeks postpartum and screening for prediabetes or type 2 diabetes every 1–3 years thereafter, with lifestyle intervention or metformin for those who screen positive.^8^ Cardiovascular surveillance is also increasingly recognized in recent years. The 2024 American Heart Association scientific statement on postpartum cardiovascular risk after adverse pregnancy outcomes, including GDM, calls for close monitoring of BP, lipids, glycemia, and weight.^9^ The kidney, by contrast, is essentially absent from this framework. No major guideline specifies surveillance of kidney function after GDM. A woman with prior GDM is therefore screened for dysglycemia at 4–12 weeks and counseled on cardiovascular risk factors, yet rarely has albuminuria checked, despite a consistent signal of kidney risk after GDM.^5^ This also stands in contrast to hypertensive disorders of pregnancy, where the long-term kidney risk is well recognized and a postpartum review of BP and kidney function is endorsed by major international societies.

While the optimal timing and frequency of subsequent monitoring warrant further study, a reasonable first step could be to add eGFR and albuminuria screening to the postpartum visit that already exists for the oral glucose tolerance test at 4–12 weeks, especially for women with recurrent GDM, insulin-treated GDM, GDM superimposed on a hypertensive disorder of pregnancy, or morbid obesity, who are at higher risk. This 4–12-week window, the so-called fourth trimester, is when women remain in close clinical contact and behavior change is most achievable, and the finding that CKD risk is already detectable within the first year postpartum^4^ lends urgency to engaging at this point. Any abnormal findings should be confirmed at 6–12 months postpartum, once gestational hyperfiltration has resolved and a true nonpregnant baseline can be established. The American Heart Association’s 2024 statement offers a usable framework for operationalizing such surveillance, anchored in lifestyle modification, including healthy diet, regular physical activity, and weight management, with the Life's Essential 8 metrics as a guide.^9^ Metformin has the strongest pharmacologic evidence in this population, reducing incident type 2 diabetes by 40% in women with prior GDM in the Diabetes Prevention Program,^10^ although its kidney and cardiovascular effects in this population are untested. A recent sodium–glucose cotransporter 2 inhibitor trial in GDM was neutral on the preservation of *β*-cell function^11^; dedicated trials with kidney and cardiovascular end points are still needed.

As the nephrology field moves toward preserving kidney health rather than merely treating established kidney disease, GDM offers a unique opportunity for early identification and potential intervention in a population of young women with substantial cumulative lifetime risk. In summary, GDM should not be read as a transient obstetric event that resolves at delivery. Rather, it is an early window into cardiovascular–kidney–metabolic risk, and the kidney deserves attention in the postpartum care of women with a history of GDM.
